# Evaluation of Corrosion and Its Impact on the Mechanical Performance of Al–Steel Joints

**DOI:** 10.3390/ma17143542

**Published:** 2024-07-17

**Authors:** Weiling Wen, Blair Carlson, Mihaela Banu

**Affiliations:** 1 Department of Mechanical Engineering, University of Michigan, Ann Arbor, MI 48109, USA; wlwen@umich.edu; 2 General Motors Research & Development, Warren, MI 48090, USA

**Keywords:** Al–steel joints, evolution of the corrosion, SPR, RSW

## Abstract

Aluminum–steel joints are increasingly used in the automotive industry to meet the requirements for energy saving and emission reduction. Among various joining technologies, self-pierce riveting (SPR) and resistance spot welding (RSW) are two well-established technologies for fabricating dissimilar joints with stable and high mechanical performance. However, corrosion will occur in these joints inevitably due to different electrochemical properties, which can degrade the surface quality and the mechanical performance, such as strength. This paper presents a method of understanding the corrosion mechanisms in joining aluminum and steel. For this understanding, a hybrid method combining experimental observations, mechanical properties identification, and analytical approaches was used to assess the evolution of the impact of corrosion on the joining performance, such as traction separation curves. The study was conducted on common combinations used in the vehicles, e.g., a 1.2 mm thickness aluminum alloy (AA 6022) and 2.0 mm thickness hot deep galvanized steel (HDG HSLA 340) joined by SPR and RSW. After the fabrication of these joints, accelerated cyclic corrosion tests of up to 104 cycles were performed, which reproduced the environmental conditions to which a vehicle was exposed. By investigating the microstructural evolution within the joints, the corrosion mechanisms of SPR and RSW joints were revealed, including the initiation and propagation. Moreover, the intrinsic impact of the corrosion on the mechanical performance, including the strength, axial stiffness, and crashworthiness, was analyzed by performing a lap-shear test. It showed that as corrosion proceeds, the fracture modes and mechanical performance are affected significantly.

## 1. Introduction

Energy has been one of the biggest global concerns due to the continuous growth of the population and industrialization. According to data from the year 2019 released by the Energy Information Administration, transportation of people and goods accounts for about 28% of all energy consumption, and petroleum products account for about 91% of the total transportation sector energy use in the U.S. [1]. The large amount of energy consumed and the greenhouse gas emissions (GHGs) created in transportation do harm to the Earth’s environment significantly, which threatens the sustainable development of human beings. In addition, the rapid development of automotive transportation in developing countries is further aggravating this critical challenge.

One promising and feasible approach is to develop a lightweight vehicle. It was reported that every 100 kg weight reduction could lead to a fuel saving of 0.5 L per 100 km and a reduction of 9 g CO_2_ per km [2]. Automotive manufacturers and researchers proposed multi-material designs to achieve lightweight vehicles while ensuring high mechanical performance, such as high-strength steel, aluminum alloys, magnesium, glass fiber reinforced thermoplastics, etc. Among various lightweight materials, aluminum alloys (Al) stand out due to low density, high relative mechanical properties, good formability, and good corrosion resistance. It has been successfully applied to some lightweight structural parts and body-in-white constructions, especially the 6xxx series [3,4,5,6]. Meanwhile, steel continues to be used in automotive body structures for crashworthiness, stiffness, and high strength. Compared with conventional high alloy steels, high-strength low alloy (HSLA) steels exhibit higher strength and better weldability and are hence widely used in automotive parts [7].

To achieve combined advantages and desired performance of automobile parts, various technologies have been developed for joining aluminum alloys to steels, including welding, laser brazing, adhesive bonding, and mechanical fastening [8,9,10,11,12]. Among these technologies, explosion joining, laser impact welding [13], self-pierce riveting (SPR) [14,15,16,17], and resistance spot welding (RSW) [17,18,19,20] stand out due to the high speed, cost efficiency, and high mechanical performance of the Al–steel joints. SPR is a cold mechanical joining process used to join two or more sheets by driving a hollow rivet through the top or middle sheets and partially piercing into the bottom sheet to form a mechanical interlock [21]. The mechanical interlock between the rivet and the base material ensures high initial stiffness and high ductility. SPR is widely used in the joining of similar materials, and in order to join Al and steel, researchers modified this technology because of the large difference in flow stress in Al and steel sheets and proposed robust process windows with regard to the geometries of the die and rivet, and the thickness combination [14,15,16,22,23,24]. RSW is a process using the electrical resistance heat between sheet interfaces to create a localized fusion zone and form a weld joint upon cooling. It is a new joining technology and increasingly attracts both automotive manufacturers and researchers due to a very short cycle time (<1 s), low cost, flexibility, and material savings in this process. The challenge of RSW that exists today in its application of joining Al to steel is the formation of brittle intermetallic compounds (IMCs), such as Fe_2_Al_5_ and FeAl_3_, at the joint interface, which will degrade the mechanical performance of the joint [25,26,27]. Miyamoto et al. [18] proposed that when the IMC layer is less than 2 µm thick, the strength is acceptable. Other researchers [25,28,29] revealed that joints show higher strength when the IMC formation is constrained when it is developed, exhibiting discontinuities. Larger weld nuggets contribute to higher strength and preferable fracture mode (button pullout) [27]. Recently, General Motors (GM) has developed a proprietary resistance spot welding process using Multi-Ring Domed (MRD) electrode geometry with acceptable mechanical performance [30], which will be further used in this paper.

Although SPR and RSW processes are successfully used to fabricate Al–steel joints with high mechanical performance, the difference in electrochemical potential of Al and steel makes the joints susceptible to galvanic corrosion. Wloka et al. [31] studied the electrochemical properties and corrosion behavior of Al–steel welded via laser brazing and showed that the degree of corrosive deterioration depends on the cathode behavior of the adjacent metal. Shi et al. [32] studied the corrosion behavior in weld brazing joints of Al and galvanized steel. It was noticed that galvanic corrosion occurs at the coupling area of the two materials and, moreover, it was found that the corrosion rate is influenced by temperature. Lim et al. [33,34] investigated the corrosion behavior of friction bit joining (FBJ) of Al and steel. It was observed that the crevice at the contact interface creates a path for a corrosive media, such as an electrolyte, which induces severe localized galvanic corrosion. However, a retard of the corrosion was obtained by applying adhesives at the overlap area. Lei et al. [35] studied the corrosion behavior of CMT spot-joined Al (Al6022) to galvanized steel (DC03) and reported that the weld strength decreases significantly with corrosion. Moreover, the separation of the joint was tested, and it was found that the fracture mode changes with corrosion from a ductile failure to an interfacial failure mode.

Among the publications studying the corrosion behavior of Al–steel joints, several researchers studied SPR Al–steel joints, and a limited number of studies addressed RSW Al–steel joints. For example, Calabrese et al. [17] studied galvanic coupling zones in SPR joints and revealed that the corrosion at the Al–steel overlapping area influenced the degradation of mechanical properties significantly. Joint configuration, especially the thickness of aluminum, plays an important role in the failure mechanism and the durability of corroded joints. Mandel and Kruger [9] studied the electrochemical properties of different components within Al–steel SPR joints and the pitting corrosion in aluminum and revealed the heaviest corrosion attack was at the end of the overlap. Kotadia et al. [36] investigated the effect of a coating on the corrosion behavior of Al–steel SPR joints and proposed a zinc–aluminum–magnesium coating to retard the rate of corrosion since the formation of Mg_2_^+^ and Al_3_^+^ ions delay the transformation of basic zinc salts to ZnO. Maddela et al. [10] compared the corrosion behavior of SPR and RSW joints based on electrochemical potentials and lap-shear strength and revealed that SPR joints are more susceptible to galvanic corrosion, and adhesive application at the overlap area helps enhance the strength after corrosion.

The above-mentioned studies agreed that strong galvanic corrosion occurs at the coupling areas between Al and steel joints, and, as a result, the strength decreases significantly. Corrective measures are proposed to delay corrosion, e.g., applying adhesives and/or coatings. However, questions still remain, such as how the corrosion evolves and which mechanisms lead to the depletion of the joint’s mechanical performance, as well as its impact on crashworthiness and stiffness behavior. Hence, in this work, an in-depth experimental and analytical analysis of the corroded SPR and RSW Al–steel joints under different corrosion durations is performed.

## 2. Experimental Procedure

### 2.1. Materials and Joining Experiments

SPR is a mechanical joining process used to join two or more sheets by piercing a rivet through the upper sheet and/or the middle sheets and flaring a skirt of the rivet into the bottom sheet to create an interlock. On the other hand, the RSW process creates metallurgical bonding at the faying interface by melting Al and/or steel under the heat generated by the electric resistance.

**Figure 1:**
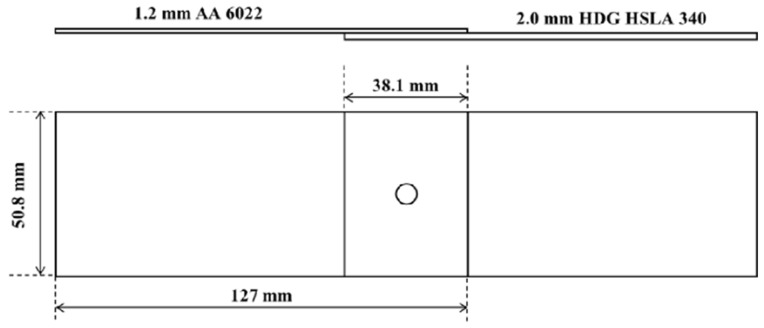
Lap-shear configuration and dimensions of the AA6022—HDG HSLA 340 joint. The circle indicate the welding spot.

SPR and RSW processes were used to join the 1.2 mm AA6022 sheet and the 2.0 mm HDG HSLA 340 sheet. Joining was performed at the General Motor Research Center. The configuration of the lap-shear joints and the dimensions of the coupons are detailed in Figure 1.

In the SPR process, countersunk rivets and domed dip dies by Henrob Co., MI, USA, were used. The rivets are made of carbon steel and coated with zinc. More details about the process are listed in Table 1. In the RSW process, Multi-Ring Domed (MRD) electrodes comprised of CuZr C1500 copper alloy were used in combination with a multiple solidification weld schedule [30]. The welding parameters are listed in Table 2.

### 2.2. Methods for an In-Depth Analysis of Corrosion Evolution

#### 2.2.1. Cyclic Corrosion Testing

The corrosion behavior of SPR and RSW joints was evaluated by cyclic corrosion testing following the standard GMW14872 [37], which reproduces the environmental conditions to which a vehicle is exposed. One corrosion cycle is defined, and it includes three phases: the first 8 h of salt spray (Ambient Stage), the second 8 h of humidity (Humid Stage), and the last 8 h of drying off (Dry-off Stage). The exposure conditions in different phases are listed in Table 3, and the composition of the salt solution is given in Table 4. The joined SPR and RSW specimens were placed at an angle of 30 degrees to the vertical reference line, with steel on the top and aluminum on the bottom. In order to have a better understanding of corrosion evolution, the duration was set as 1 cycle, 7 cycles, 14 cycles, 26 cycles, 48 cycles, 72 cycles, and 104 cycles.

#### 2.2.2. As-Received Material Electrochemical Response

The electrochemical properties of each component (Al substrate, HDG, steel substrate, rivet) in the joints were studied by the open circuit potential (OCP) and the dynamic polarization in 3.5%wt sodium chloride solution. Sample sheets (AA 6022, HDG HSLA 340, and bare HSLA 340) and a mounted rivet in resin were washed with distilled water and acetone and then dried before the testing. All the electrochemical measurements were conducted on the Solartron SI1280, MI, USA Electrochemical measurement unit. A standard flat cell was used, consisting of a saturated calomel reference electrode (RE), a pure Pt-mesh counter electrode (CE), and the working electrode (WE), which is the sample itself. The contact area between the sample sheets (AA 6022, HDG HSLA 340, and bare HSLA 340) and electrolyte (WE area) is 1 cm^2^ (standard), while the contact area between the mounted rivet is the rivet head area measured. Before the dynamic polarization, 3600 s of OCP was conducted to reach a stable potential, and the scan rate in dynamic polarization was set at 1 mV/s.

#### 2.2.3. Strain-Induced Electrochemical Response

Considering that joining processes induce a level of plastic strain (e.g., in RSW, the ring has periodic indentations, and in SPR, the rivet plunges the sheets), the effect of plastic deformation on the corrosion properties was also investigated. The plastic strain was induced in bare AA 6022 and HSLA 340 sheets by thickness reduction through a rolling mill. The thickness of AA 6022 was reduced from 1.2 mm to 0.7 mm (thickness reduction of 41.7%), while the thickness of HSLA 340 was reduced from 1.6 mm and 0.8 mm (thickness reduction of 20% and 60%, respectively). Considering that cold rolling is a plane strain process, the longitudinal strain is equivalent to the nominal strain (thickness reduction). The setting of the rolling mill is listed in Table 5. After the rolling experiments, OCP was performed on these deformed Al and steel sheets.

#### 2.2.4. Experimental Analysis

Lap-shear tests were conducted for SPR and RSW Al–steel joints before and after corrosion (1 cycle, 7 cycles, 14 cycles, 26 cycles, 48 cycles, 72 cycles, and 104 cycles) on the Instron 33R 4469, MI, USA. The head speed was set at 2 mm/min, and the aluminum was pulled out of the steel. Each test was repeated 5 times to maintain the consistency of the data.

An optical microscope and scanning electron microscope (SEM, TESCAN MIRA3 FEG-SEM, MI, USA) were employed to reveal the microstructural evolution with corrosion in the joints before and after corrosion exposure. Samples were cross-sectioned at the center, cold mounted, and then ground and fine-polished to 0.06 microns. After polishing, samples were rinsed with distilled water and acetone and dried in the air at room temperature. Three samples for each cycle were polished and then examined.

## 3. Results and Discussion

### 3.1. Electrochemical Measurements

**Figure 2:**
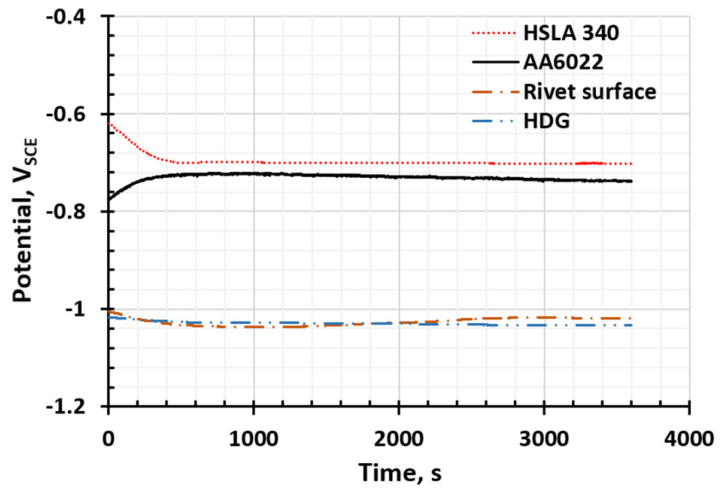
OCP curves of the components in the Al–steel SPR and RSW joints.

Following electrochemical measurements, OCP curves of different components in the SPR and RSW joints were plotted and analyzed (Figure 2). HDG (the coating on the steel surface) has the lowest electrochemical potential, −1.03 VSCE, and the SPR rivet has almost similar potential as HDG, −1.02 VSCE. AA 6022 has a lower potential, −0.74 VSCE, and bare HSLA 340 has the lowest potential, −0.70 VSCE. According to the potentials, HDG and the rivet are the most active and more susceptible to corrosion attack, followed by AA 6022. Bare HSLA 340 is the most noble material in the joints.

**Figure 3:**
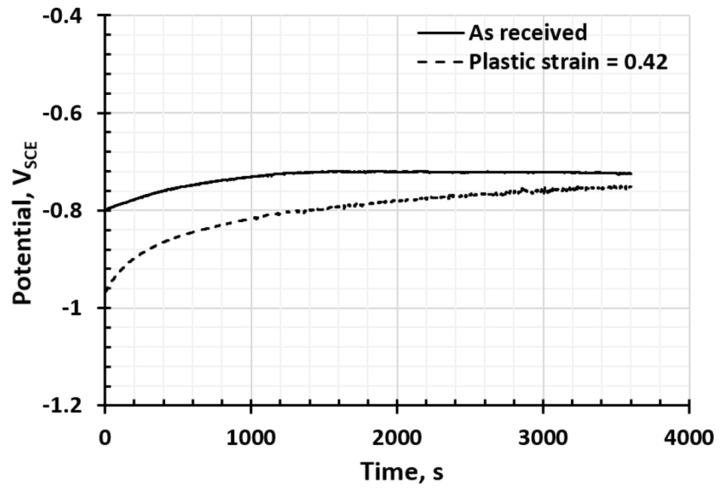
The effect of plastic deformation on the OCP of AA 6022.

**Figure 4:**
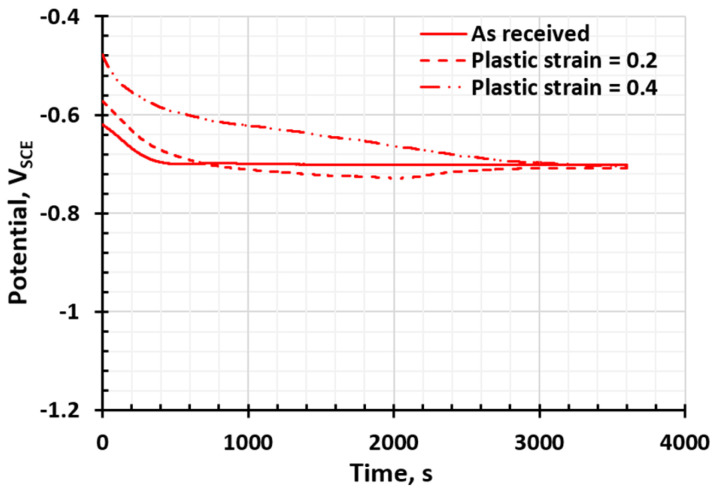
The effect of plastic deformation on the OCP of HSLA 340.

Figure 3 and Figure 4 show the electrochemical potential of AA 6022 and HSLA 340 with different levels of plastic strain, respectively. According to Figure 3, with some plastic strain (0.42), the electrical potential decreased from −0.74 V_SCE_ to −0.77 V_SCE_, indicating that the deformed AA 6022 is more active and prone to corroding. Other researchers also reported that the presence of plastic strain increases the susceptibility to corrosion and the corrosion rate in different aluminum alloys. It is revealed that a higher driving force of micro-galvanic corrosion potential (such as Mg_2_Si-Al matrix) [38], and decreased charge transfer magnitude (increased corrosion rate) under plastic strain [39]. For HSLA 340, there is no statistical change in the corrosion potential for steel with/without plastic strain, as shown in Figure 4; it stays around −0.70 V_SCE_. However, even if there are only small changes in the measured corrosion potential, the plastic strain affects the dynamic corrosion rate. This conclusion was confirmed by Cui et al. [40] and Xu et al. [41], who stated that the corrosion rate of steel increases as strain is imposed, and this phenomenon is owed to the increase in active sites and the surface roughness [40].

### 3.2. Corrosion Behavior of Al–Steel Joints

#### 3.2.1. SPR Al–Steel Joints

**Figure 5:**
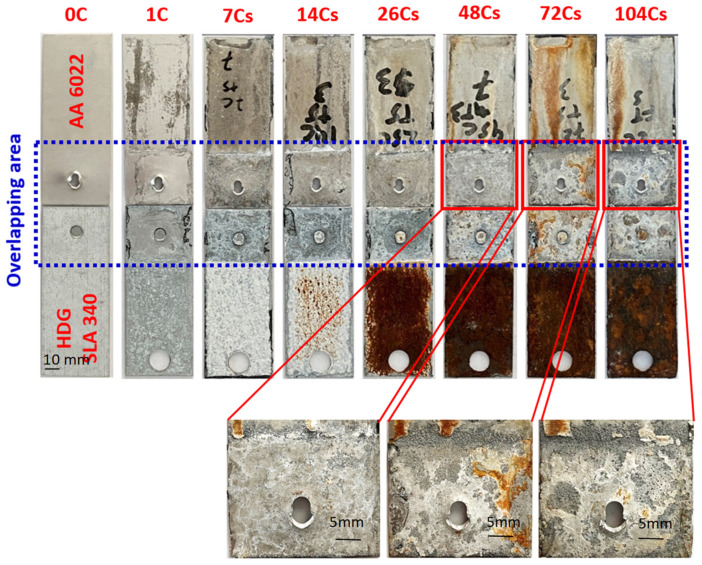
Fractured SPR joints after different cycles of corrosion.

**Figure 6:**
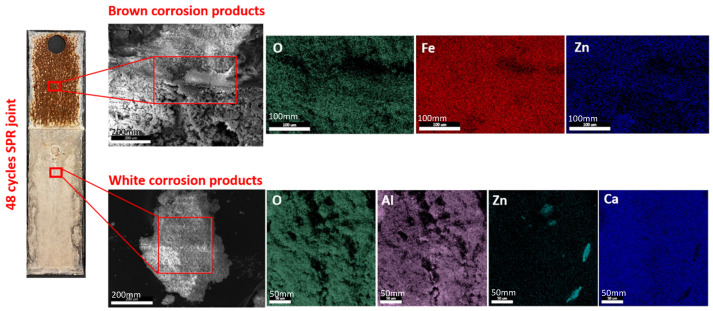
EDX mapping of surface corrosion products.

The overall corrosion conditions of the SPR joints were optically examined after the lap-shear tests were applied to coupons before and after corrosion, as shown in Figure 5. A visual analysis of the corroded sample indicates two types of corrosion products that are formed during corrosion. The corrosion products on the surfaces were examined by performing EDX analysis, as shown in Figure 6. According to the mapping results, the white products on the aluminum surface mainly consist of zinc oxides and aluminum oxides. Meanwhile, the brown products are mainly comprised of iron oxide and zinc oxides.

The white corrosion products start to appear and aggregate on the steel surface after 1 cycle, and then brown corrosion products are generated after 14 cycles. As the corrosion duration increases, the layer of brown corrosion products becomes thicker. The change in steel is noticeable, compared to the changes of the AA 6022, which became dull gray after 1 cycle, demonstrating that the aluminum has been oxidized and has been covered by some white corrosion products after 14 cycles. Hence, the white corrosion products covering the steel surface initially are the zinc oxides, which are the result of the corrosion of HDG. When the steel substrate was exposed and corroded (after 14 cycles), brown iron oxides were generated. Zinc oxides and iron oxides appeared on the aluminum surface with longer corrosion exposure, which came from the steel surface since the joints were leaned on the holder in the test chamber with steel on the top. Moreover, within the Al–steel overlapping area, it was observed that AA 6022 was corroded, and the most affected zone was at the overlap end of Al, while the steel was protected. After 48 cycles, pits started to be observed at the overlap end of Al. As corrosion proceeded, the pit area spread into the joints, and at 104 cycles, the whole piece of Al at the overlapping area became severely porous. According to the OCP measurements, the electrochemical potential of HDG is −1.03 V_SCE_, AA 6022 is −0.74 V_SCE,_ and bare HSLA 340 is −0.70 V_SCE_. Hence, at the overlapping area, HDG is corroded when the salt solution penetrates into the crevice. When HSLA 340 is exposed, and the salt solution is present, a galvanic cell is generated, AA 6022 is corroded, and HSLA 340 is protected.

**Figure 7:**
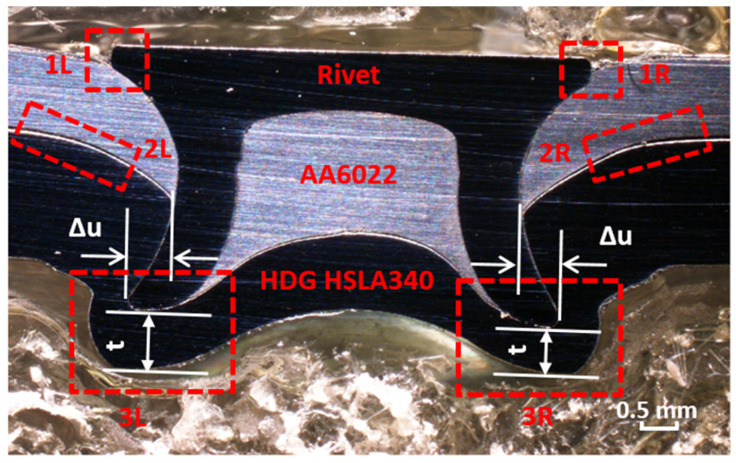
The cross-section of the SPR joint before corrosion and three possible paths for corrosion to proceed.

In order to reveal the corrosion evolution within the joints, OM analyses were performed. The cross-section of SPR joints before corrosion is shown in Figure 7. The interlock (Δu) and bottom thickness (t) are 0.573 mm and 0.730 mm, respectively. Three paths exist that corrosion will proceed into the joints: one is at the Al–rivet crevice (site 1: 1L and 1R), the Al–steel crevice (site 2: 2L and 2R), and steel (site 3: 3L and 3R), as shown in Figure 7.

**Figure 8:**
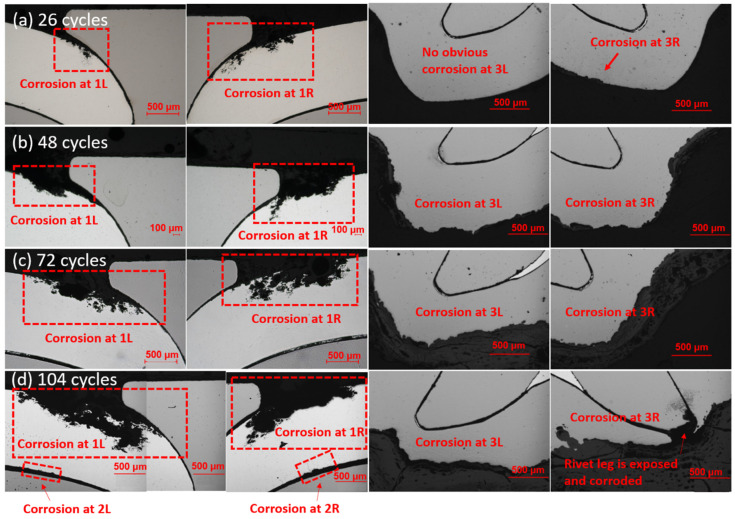
Microstructural evolution of SPR Al–steel joints under cyclic corrosion. (**a**) 26 cycles; (**b**) 48 cycles; (**c**) 72 cycles; (**d**) 104 cycles.

**Figure 9:**
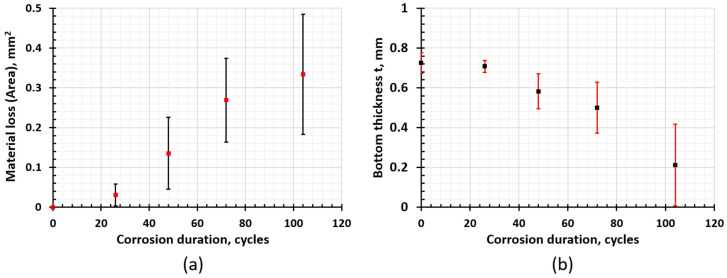
(**a**) Evolution of material loss in AA 6022 at site 1 with different corrosion exposure. (**b**) Evolution of t (bottom thickness defined in Figure 7) at site 3 with different corrosion exposure times. The plot indicates the minimum, the maximum (black) and the mean (red) for each measurement for material loss and the minimum, the maximum (red) and the mean (black) for each measurement for the thickness t.

**Figure 10:**
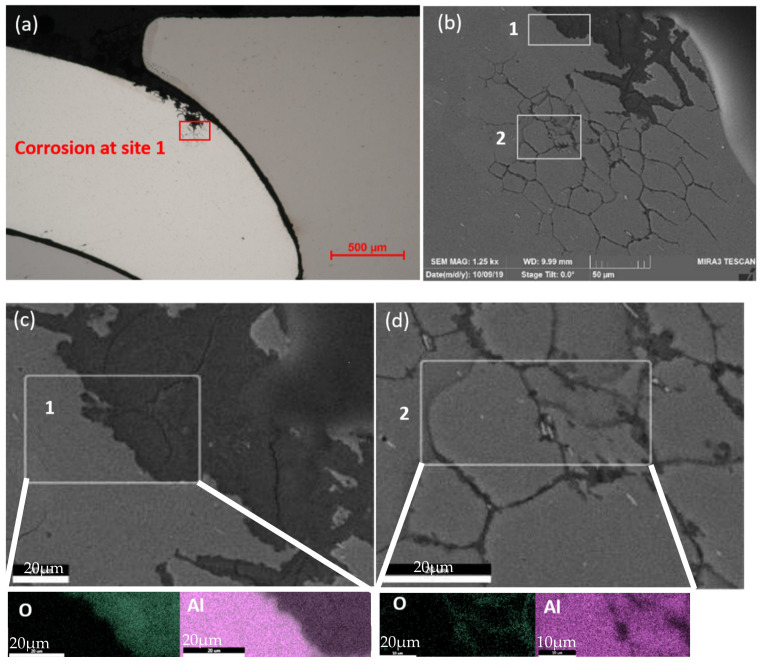
EDX analysis of the IGC in AA 6022 of 26 cycles SPR joint; (**a**) corrosion installed at the interface between the upper coupon and the rivet; (**b**) details of the corrosion initiated at site 1 degradation of the grain boundaries; (**c**) intragranular corrosion caused by material loss; (**d**) corrosion propagation through advancing grain boundary degradation. Below (**c**,**d**) EDX analysis of the indicated areas 1 from (**c**) and 2 from (**d**) showing the volume Oxygen (O) presence and Aluminum (Al).

Figure 8 shows the evolution of corrosion in Al–steel SPR joints under cyclic corrosion. Obvious corrosion is first observed at site 1 in 26 cycles. In the 26 cycles samples, multiple localized corrosion initiates in AA 6022 along the Al–rivet interface. These shallow and small initiations expand and evolve into wider and deeper localized corrosion with a much higher aspect ratio, as shown in Figure 8a–d. No obvious corrosion is observed at site 2, and only slight corrosion is observed up to 104 cycles at site 2, as shown in Figure 8d. Though AA 6022 at the overlap became porous due to the galvanic corrosion, the crevice at site 2 is small enough to prevent the salt solution from penetrating into the joints and, hence, delay the corrosion here. In site 3, as the steel is corroded, the bottom thickness decreases, and the rivet is even exposed and corroded in one of the 104 cycle samples, which reduces the interlock and, hence, the strength significantly. In order to quantify the corrosion rate, material loss at site 1 and the bottom thickness at site 3 were measured by Image J version 1.40j and plotted in Figure 9a,b, respectively. The variation became larger as corrosion proceeded. According to Figure 9, material loss in AA6022 increased significantly after 26 cycles, and the decreasing rate reduced after 72 cycles, indicating a slower corrosion rate. This is because thick corrosion products like aluminum oxides prevent the salt solution from penetrating into the remaining aluminum. The bottom thickness decreased after 26 cycles, and the decreasing rate increased significantly from 72 cycles to 104 cycles, as shown in Figure 10, indicating a higher corrosion rate. As discussed in Section 3.1, mechanical deformation will increase the corrosion kinetics [42] by decreasing the electron work function [43], which is the energy required to withdraw an electron completely from a metal surface, the electrochemical activity, active sites, and surface roughness [40], etc. Compared with aluminum oxides, iron oxides are looser, and salt solution penetrates the remaining steel continuously. Therefore, the corrosion at site 3 is the most dangerous, and in the foreseeable future, the rivet will be corroded, and the interlock will decrease; hence, the joint strength will reduce significantly.

Microscopic observations showed that intergranular corrosion (IGC) was observed in AA 6022 corroded coupons. EDX analysis was performed for the 26-cycle joints. Two locations in AA 6022 along the Al–rivet interface were investigated, as shown in Figure 10. In area 1, the black zone mainly contains oxygen, indicating Al has been completely corroded away. In area 2, Al exists in the grain matrix while oxygen exists in the grain boundaries, indicating the material in the grain boundaries has been corroded while the grain matrix has not. It is the typical propagation pattern of IGC that corrosion propagates through the grain boundaries since the bulk of grains are more electrochemical noble than that of grain boundaries; grains are then “removed” without the bond. In fact, the IGC susceptibility of 6xxx Al alloys is affected by the ratio of Mg/Si, Cu content, the depletion of Si and Cu atoms, precipitation of Si and Cu phases at the grain boundaries, and the preferential dissolution of Mg_2_Si phases [44,45].

#### 3.2.2. RSW Al–Steel Joints

**Figure 11:**
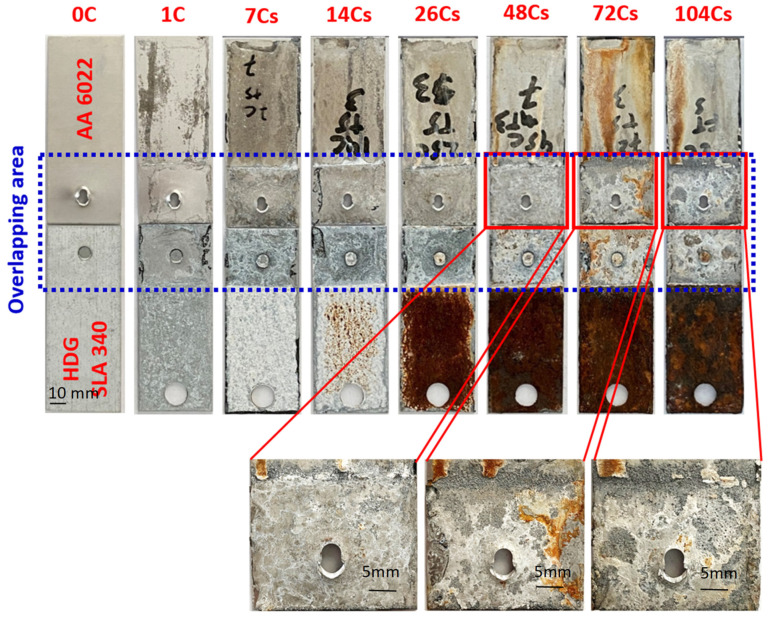
Fractured RSW joints after different cycles of corrosion.

The overall corrosion of RSW joints with different corrosion durations after the lap-shear test is shown in Figure 11. The corrosion on the surfaces of Al and steel, which are out of the overlapping area, is similar to that in SPR joints. For 104 cycles, the joints have already debonded before being carried out. Obvious pits are also observed in AA 6022 at the overlapping area after 26 cycles. As corrosion proceeds, the pitting area expands towards the joint, and up to 104 cycles, the whole piece of AA 6022 at the overlapping area becomes porous.

**Figure 12:**
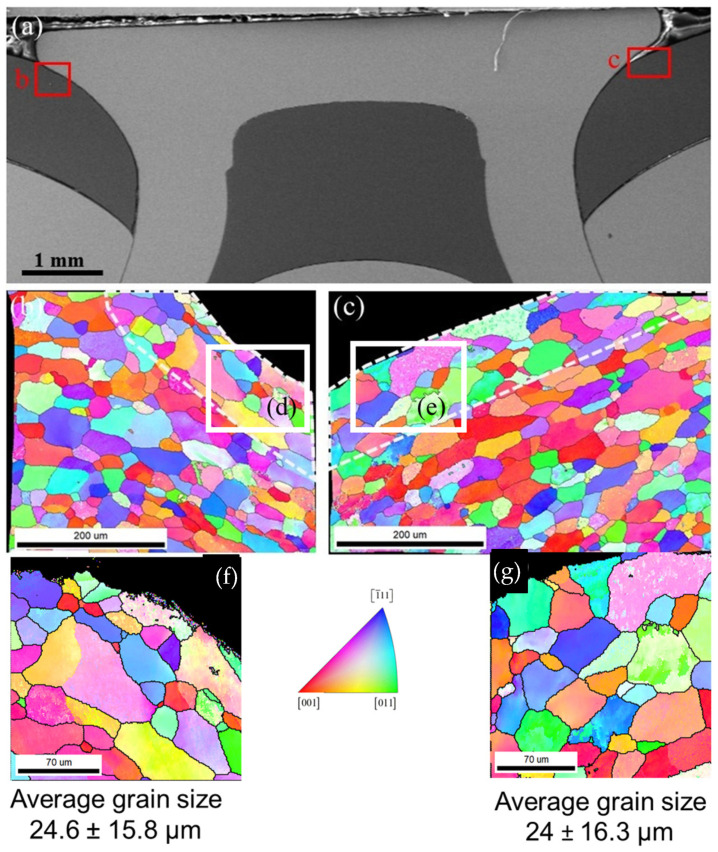
EBSD analysis in AA 6022 along the Al–rivet interface using 30 kV 20BI step size 1 μm. (**a**) Secondary electron micrograph of SPR joints showing the locations of the measurements; (**b**,**c**) inverse pole figure orientation maps with respect to sample normal, taken from regions b and c shown in (**a**), respectively, with high angle grain boundaries (misorientation angle > 10°) delineated in white lines; (**d**,**e**) show grain size uniformity in the delineated white lines; (**f**,**g**) show magnified grain distribution at the interface with the rivet (left and right).

Figure 12 shows the EBSD analysis in Al at site 1 before corrosion. The grains at this site are elongated along the contour of the rivet; the closer to the rivet the Al material is, the more obvious the elongation is (see the grains marked by dashed white lines in Figure 12b,c). According to Figure 8, shallow and small corrosion initiations evolve into wider and deeper localized corrosion of a much higher aspect ratio. It is clear that the IGC propagates much more profoundly through the elongation direction.

**Figure 13:**
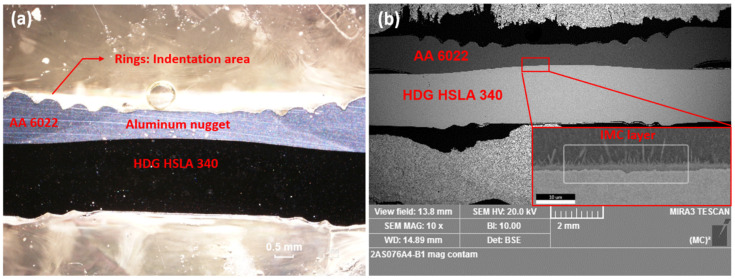
The cross-section of RSW joints before corrosion under (**a**) OM and (**b**) SEM.

Figure 12 shows the cross-section of RSW joints before corrosion. Al and steel are joined by a diffusion layer at the faying interface, which consists of brittle intermetallic compounds (IMCs). The flat-shape IMC is Fe_2_Al_5_, and the needle-shape one is FeAl_3_ [19]. The cross-section of joints under different corrosion durations was examined by OM, and corrosion was first observed on the rings (electrode indentation area) of AA 6022 for 14 cycles, as shown in Figure 13. Corrosion initiates here because of higher surface roughness and mechanical deformation. According to Figure 13, material loss in this area increased significantly from 14 cycles to 48 cycles; however, there is not much difference between samples from 48 cycles and 72 cycles. It is because the corrosion products (aluminum oxides) covered on the surface help prevent salt solution from penetrating into the remaining AA 6022, and hence the corrosion rate decreased.

**Figure 14:**
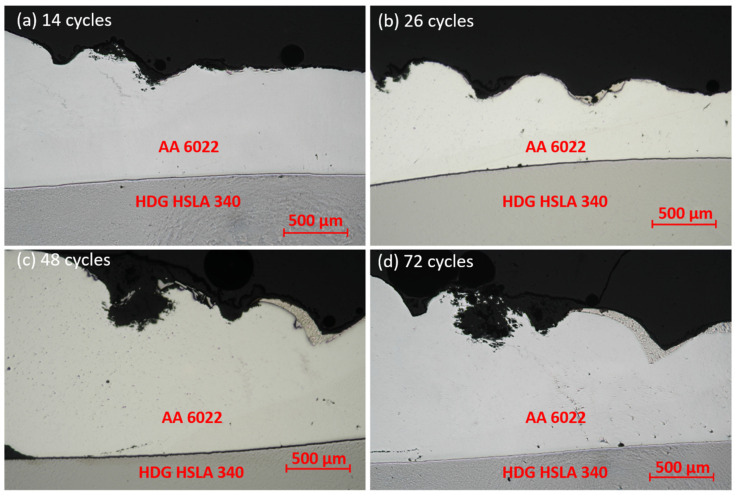
SPR localized corrosion on the rings (the electrode indentation area) of AA 6022 with (**a**) 14 cycles of corrosion, (**b**) 26 cycles of corrosion, (**c**) 48 cycles of corrosion, and (**d**) 72 cycles of corrosion.

**Figure 15:**
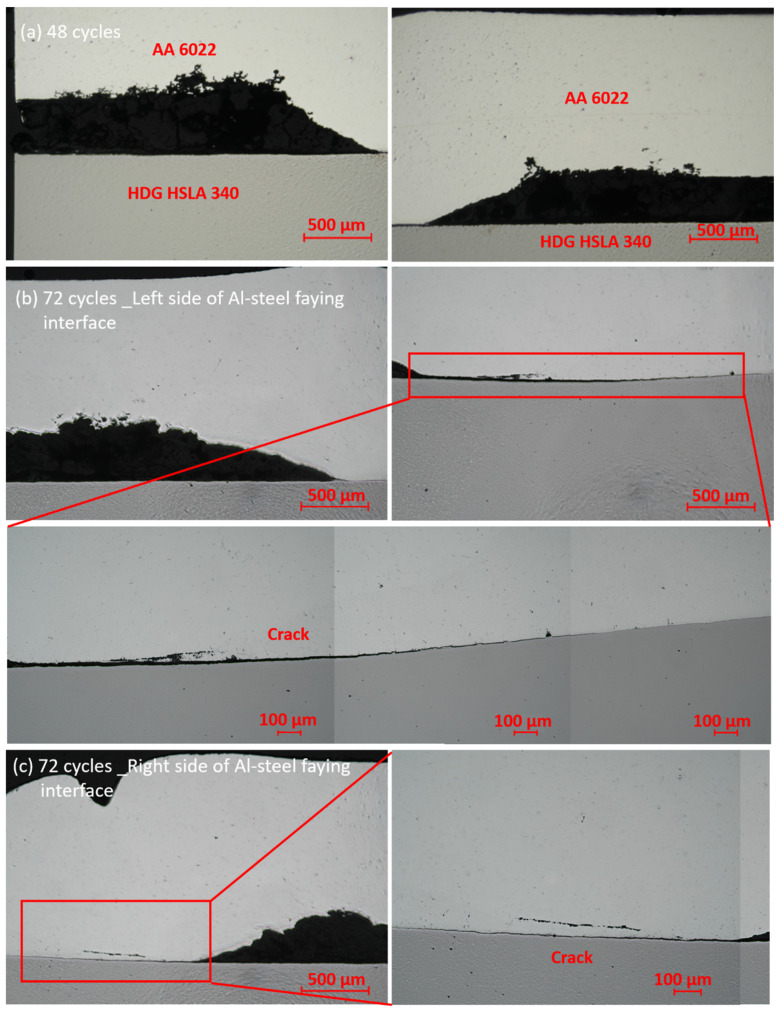
RSW Corrosion evolution along the Al–steel interface: (**a**) 48 cycles joint; (**b**) left side of 72 cycles joint; (**c**) right side of 72 cycles joint.

With longer exposure in a corrosive environment, another localized corrosion was observed at the Al–steel crevice after 48 cycles, as shown in Figure 14a. Since AA 6022 is more active than HSLA 340, AA 6022 is corroded, and HSLA 340 is protected in the overlapping area. As the duration increases, the corrosion proceeds to the IMC layer, as shown in Figure 14b. Cracks are formed on two sides. When it comes to 104 cycles, the crack propagates to the whole layer of IMCs, and as a consequence, the joint fails. It is reported that the OCP of Al_3_Fe and Fe_2_Al_5_ are −0.382 V_SCE_ in 0.6 M NaCl solution [46] and −0.5661 V_SCE_ in 3.5%wt NaCl solution [46,47], respectively. Both of them are more noble than AA 6022. In other words, the joints’ failure owes to the stress corrosion cracking (SSC) instead of the IMC layer being corroded away. Similar it is presented in Figure 15 for RSW.

### 3.3. The Impact of Corrosion on the Mechanical Performance

The mechanical performance of SPR and RSW joints before and after corrosion was evaluated by lap-shear testing with regard to the strength, axial stiffness, the energy absorbed at failure, and fracture modes.

**Figure 16:**
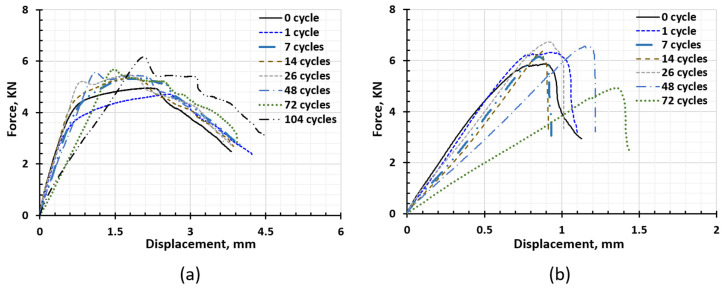
Evolution of the F-D curves under cyclic corrosion of (**a**) SPR joint and (**b**) RSW joint.

The force and displacement (F-D) curves of SPR and RSW are shown in Figure 16a,b, respectively. For SPR joints, the F-D curves tend to be taller and slimmer as the corrosion duration increases, indicating the fracture mode turns ductile into a more brittle fracture. “Hills” were also observed in the curve after 26 cycles, and as the corrosion duration increased, the “hill” became taller and steeper. This was because the brittle corrosion products became thicker and tightened the joints. When they break under loading, the force drops significantly. Similarly, the F-D curves of RSW become steeper after seven cycles, implying the fracture mode turns to become more brittle.

**Figure 17:**
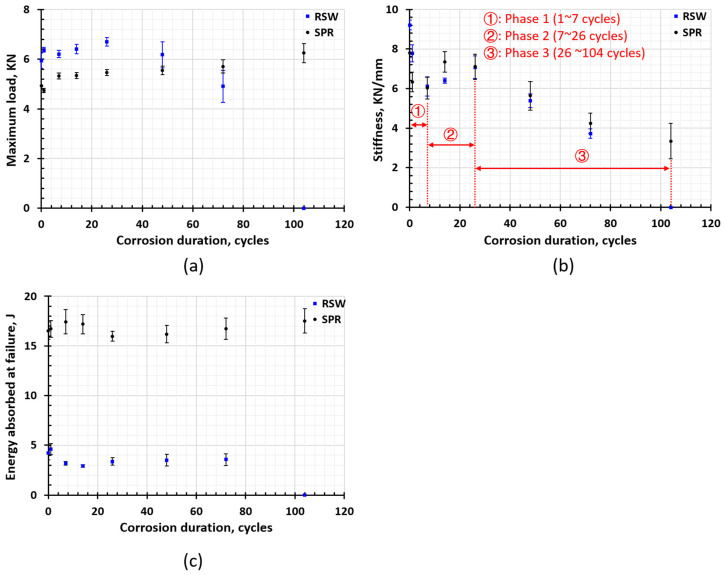
(**a**) Evolution of maximum load under cyclic corrosion; (**b**) evolution of stiffness under cyclic corrosion; (**c**) evolution of energy absorbed at failure under cyclic corrosion.

The strength of non-corroded and corroded Al–steel SPR and RSW joints are plotted in Figure 17a. The average strength of both SPR and RSW joints increases slightly as the corrosion proceeds instead of decreasing. In SPR joints, galvanic corrosion attacks AA6022 at the Al–rivet head interface and the interlock is not affected until 104 cycles. In fact, the rivet leg is only exposed and corroded in one sample of 104-cycle joints. As a result, the strength does not decrease. However, the corrosion products are not stable and easily become loosened and removed from the joints during the service, which will lead to a significant decrease in strength. For RSW joints, the average strength decreases significantly since the corrosion proceeds into the Al–steel faying interface within the joint, and SCC occurs in the IMC layer after 48 cycles. Once the whole layer of IMCs cracks, the joint fails to bear any loading, and the strength drops to 0 (104 cycles). According to the above analysis, the SPR joint performs much better in retaining strength than the RSW joint does when exposed to a corrosive environment.

The strength, stiffness, and energy absorbed at failure were extracted from the F-D curve. The stiffness is obtained by fitting the data within the range of 0~2 KN, and the energy absorbed at failure was calculated by (k=F/x), where $D_{i}$ and $F_{i}$ are the force and displacement of the ith set of experimental data, respectively.

The energy to failure is calculated based on Equation (1):

(1) $$E=\sum_{i}\frac{\left(F_{i+1}+F_{i})\cdot (x_{i+1}-x_{i}\right)}{2}$$

where $F_{i}$ and $x_{i}$ denote the force and displacement of the ith set of experimental data, respectively. The energy to failure of non-corroded and corroded Al–steel SPR and RSW joints are plotted in Figure 17c. For SPR joints, the energy does not show too much difference as corrosion proceeds. This is because though the shape of the curves becomes slim, the elastic slope decreases, and the maximum load increases due to the corrosion products tightening the joint; therefore, the energy absorbed till failure does not change too much. For RSW joints, the energy increases slightly at one cycle and drops drastically from one cycle to seven cycles. Since then, it has not changed significantly and finally drops to 0 at 104 cycles. This is in accordance with the shape of the F-D curves. In one cycle, the stiffness and the extension do not change significantly, but strength increases due to the tightening effect of the corrosion products. From 7 to 14 cycles, the stiffness decreases, but the extension does not change; hence, the energy drops. The stiffness continues to decrease, but the extension increases a lot during 14~72 cycles. At some specific time between 72 cycles and 104 cycles, joints were debonded due to SCC propagating to the whole layer of the IMCs.

Therefore, SPR joints perform better than RSW joints regarding the energy to failure, especially under long-term corrosion.

The comparison of the strength, stiffness, and energy of SPR and RSW joints before and after corrosion is shown in Figure 17a–c, respectively.

According to Figure 17a, the average strength of SPR joints increases slightly as the corrosion proceeds instead of decreasing. This is because the corrosion products generated tightened the mechanical interlocking [36]. Some bonding marks were observed in the Al–steel overlapping area after fracture; the lap-shear test also helped to prove this, as shown in Figure 5. According to Figure 8, galvanic corrosion attacks AA 6022 at the Al–rivet head interface, and the interlock was not affected until 104 cycles (only in one 104-cycle sample was the rivet observed to be exposed and corroded). As a result, the strength did not decrease. However, in the foreseeable future, as corrosion continues, the interlock will decrease, and hence the strength will be reduced. For RSW joints, the average strength increases slightly from 1 cycle to 26 cycles as well. However, after 48 cycles of corrosion, the strength decreases significantly since corrosion proceeds into the IMC layer and causes SSC. Once the whole layer of IMCs cracks, the joints fail to bear any loading, and strength drops to 0 (104 cycles).

The stiffness changes of SPR and RSW show a similar trend, which can be divided into 3 phases, as shown in Figure 17b. Phase 1 is from one cycle to seven cycles, and in this phase, stiffness decreases. Phase 2 is from 7 cycles to 26 cycles, in which stiffness increases. After 26 cycles, the stiffness decreases significantly, which is phase 3.

In phase 1, very slight corrosion occurs on the surface, as shown in Figure 5 and Figure 12. It is supposed not to affect the mechanical performance significantly. The decreasing stiffness might be due to the residual stress relaxation under the elevated temperature in the Humid Stage and Dry-off Stage in the cyclic corrosion test. To validate the hypothesis, four as-received SPR joints were heated up, following the elevated temperature history in one cycle (49 °C for 8 h and 60 °C for another 8 h), and then a lap-shear test was performed. The stiffness of the four heated joints is 6.602 KN/mm, 6.876 KN/mm, 6.518 KN/mm, and 6.823 KN/mm, respectively. Compared with the stiffness of the as-received joints, which is 7.812 ± 0.173 KN/mm, the stiffness of the heated joints decreases significantly and is comparable with that of one cycle joints, 6.332 ± 0.496 KN/mm. Therefore, the stiffness decreases because residual stress is released under elevated temperature. According to Figure 17b, the stiffness continues to decrease from one cycle to seven cycles, indicating that the residual stress was not released completely until seven cycles.

**Figure 18:**
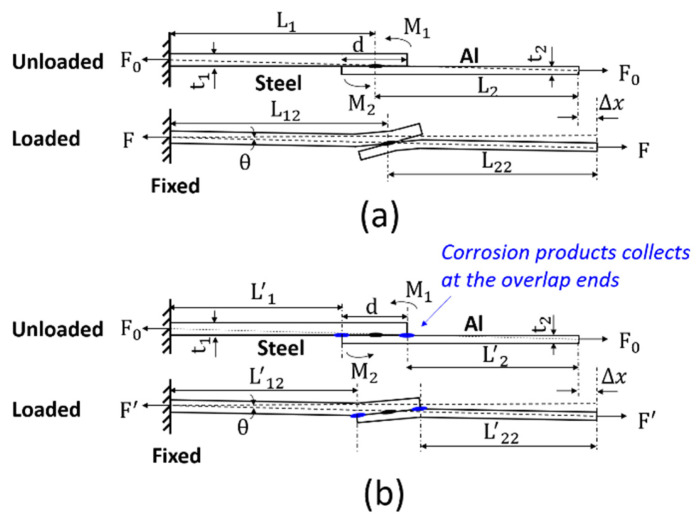
The schematic of the deformation in the lap-shear test: (**a**) Joint without corrosion products and (**b**) joint with corrosion products collected at the overlapping end.

In phase 2, from 7 cycles to 26 cycles, HDG was corroded away, and in 14 cycles, steel started to be exposed and was completely exposed in 26 cycles, according to Figure 5 and Figure 14. The corrosion products (zinc oxide mainly) and a great amount of corrosive solution solids collect at the overlap ends. For one thing, galvanic corrosion at the overlap starts here; for another thing, the joints were leaned on the holder in the corrosion chamber, and corrosion products and corrosive solution flowed to and then collected here. Under lap-shear loading, these solids acted as an “adhesive” and provided extra resistance to shearing and peeling. The schematics of the joints without/with corrosion products, which are collected at the overlapping area in the lap-shear test, are shown in Figure 18a,b, respectively. Because of the eccentricity of the forces on the fixed end and moving end, moment will be generated in both as received and corroded joints. The moment acted on the steel and Al is shown in Equations (2) and (3), respectively:

(2) $$M_{1}=F_{0}\cdot \frac{t_{1}}{2}$$

(3) $$M_{2}=F_{0}\cdot \frac{t_{2}}{2}$$

where $F_{0}$ is the clamping force before loading, $t_{1}$ is the thickness of steel, and $t_{2}$ is the thickness of Al.

Under this moment $M_{1}$ and $M_{2}$, steel and Al start to bend, as shown in Figure 18a,b. Because the corrosion products collect at the overlap ends, the material at the overlap area gains extra strength to resist deformation, and the material will bend more slightly in the corroded joint than in the as-received joint. During the lap-shear testing, the movement of the crosshead is controlled by the displacement (moving speed is 2 mm/min), therefore after some certain time t, the displacement Δx of as-received and corroded joints are equivalent. For as-received joints,

(4) $$\Delta x=(L_{12}\cdot \cos\theta -L_{1})+(L_{22}\cdot \cos\theta -L_{2})$$

where $L_{1}$ and $L_{12}$ are the length of steel from the fixed end to the center of the joint at the beginning and after a certain time t, respectively, $L_{2}$ and $L_{22}$ are the length of Al from the moving end to the center of the joint at the beginning and after a certain time t, respectively, and θ is the rotation angle of material from the fixed end to the moving end.

When the force is aligned, θ has a maximum value that:

(5) sinθmax=t1+t22L11+L22<t1+t22L1+L2=1.2100=0.016

therefore, $\theta_{\max}$ is smaller than 0.92° and cosθ is small enough to be neglected. In this case, for as-received joints, Δx can be reduced into

(6) $$\Delta x=(L_{12}-L_{1})+(L_{22}-L_{2})$$

Similarly, for corroded joints

(7) $$\Delta x=({L^{\prime }}_{12}-{L^{\prime }}_{1})+({L^{\prime }}_{22}-{L^{\prime }}_{2})$$

where ${L^{\prime }}_{1}$ and ${L^{\prime }}_{12}$ are the length of steel from the fixed end to the left overlap end before loading and after some certain time t when corrosion products collect (including corrosive solution solids) at the overlap end, and ${L^{\prime }}_{2}$ and ${L^{\prime }}_{22}$ are the length of Al from the moving end to the right overlap end before loading and after some certain time t when corrosion products collect at the overlap end. According to Equation (7), the deformation of the material in the overlap area does not contribute to Δx; it is because corrosion products collect at two ends, which act as “adhesive”; the material in the overlap area gains extra strength to resist deformation.

To obtain a specific displacement Δx, the force applied to the as-received joint F and the corroded one $F^{\prime }$ are shown in Equations (8) and (9), respectively:

(8) $$F=\frac{L_{12}-L_{1}}{L_{1}}\cdot A_{\mathrm{steel}}\cdot E_{\mathrm{steel}}+\frac{L_{22}-L_{2}}{L_{2}}\cdot A_{\mathrm{Al}}\cdot E_{\mathrm{Al}}$$

(9) $$F^{\prime }=\frac{{L^{\prime }}_{12}-{L^{\prime }}_{1}}{{L^{\prime }}_{1}}\cdot A_{\mathrm{steel}}\cdot E_{\mathrm{steel}}+\frac{{L^{\prime }}_{22}-{L^{\prime }}_{2}}{{L^{\prime }}_{2}}\cdot A_{\mathrm{Al}}\cdot E_{\mathrm{Al}}$$

where $A_{\mathrm{steel}}$ and $A_{\mathrm{Al}}$ are the cross-section area of steel and Al, respectively, and $E_{\mathrm{steel}}$ and $E_{\mathrm{Al}}$ are the Young’s modulus of steel and Al, respectively.

Since the displacement of the as-received joint and corroded joint after some certain time t is equivalent, hence $\left(L_{12}-L_{1})+(L_{22}-L_{2})=({L^{\prime }}_{12}-{L^{\prime }}_{1})+({L^{\prime }}_{22}-{L^{\prime }}_{2}\right)$. For simplicity, the portion of the deformation of Al and steel are considered as the same in two conditions, that is $L_{12}-L_{1}={L^{\prime }}_{12}-{L^{\prime }}_{1}$, $L_{22}-L_{2}={L^{\prime }}_{22}-{L^{\prime }}_{2}$. Substituting this relationship in Equations (8) and (9), $F^{\prime }>F$ since ${L^{\prime }}_{1}<L_{1}$, ${L^{\prime }}_{2}<L_{2}$. Therefore, the stiffness of the joint in which corrosion solids collect at the overlap ends is greater than that in the as-received joint ($k^{\prime }=\frac{F^{\prime }}{\Delta x}>k=\frac{F}{\Delta x}$).

It is also noted that the stiffness of SPR joints increased faster than that of RSW joints from 7 to 14 cycles and then kept almost the same from 14 to 26 cycles, while the stiffness of RSW joints continues to increase. This is because the smaller gap of SPR joints at the Al–steel interface prevents the corrosion products from flowing into the crevice, resulting in more corrosion products collecting at the overlap end. It costs RSW joints more time to collect the same amount of corrosion products since a larger gap exists along the Al–steel interface.

In phase 3 (26~104 cycles), HDG has been corroded away. Without the protection of HDG, steel starts to corrode significantly. Meanwhile, AA 6022 was under severe galvanic corrosion at the overlapping area. AA 6022 became highly porous at the overlapping ends, as shown in Figure 5 and Figure 14. These large amounts of material loss, especially the large number of pits generated in AA 6022 at overlap ends, contribute to the decrease in stiffness. According to Figure 17b, the decreasing rate of the stiffness in RSW joints is much higher than that in SPR joints, which implies more severe galvanic corrosion occurs at the overlap area.

Figure 17c shows the energy absorbed at the failure of SPR and RSW joints before and after corrosion. For SPR joints, the energy does not show too much difference as corrosion proceeds. This is because though the shape of the curve becomes slim and stiffness decreases, the peak load increases due to the corrosion products tightening the joints a little bit; therefore, the energy absorbed does not change too much. For RSW joints, the energy increased slightly at one cycle and dropped drastically from one cycle to seven cycles. Since then, it has not changed too much and finally drops to 0 at 104 cycles. It is in accordance with the shape of the F-D curves. At one cycle, the stiffness and the extension do not change significantly, but strength increases due to the tightening effect of the corrosion products. For 7~14 cycles, the stiffness decreases, but the extension does not change; hence, the energy drops. After 14 cycles to 72 cycles, the stiffness continues to decrease, but the extension increases a lot. For 104 cycles, joints were debonded due to SSC and lost any load-bearing capacity.

Compared to the corrosion behavior and the mechanical performance of corroded SPR and RSW joints, SPR joints retain the mechanical properties much better than RSW joints, including the strength, stiffness, and energy absorbed at failure.

### 3.4. Corrosion Mechanisms

**Figure 19:**
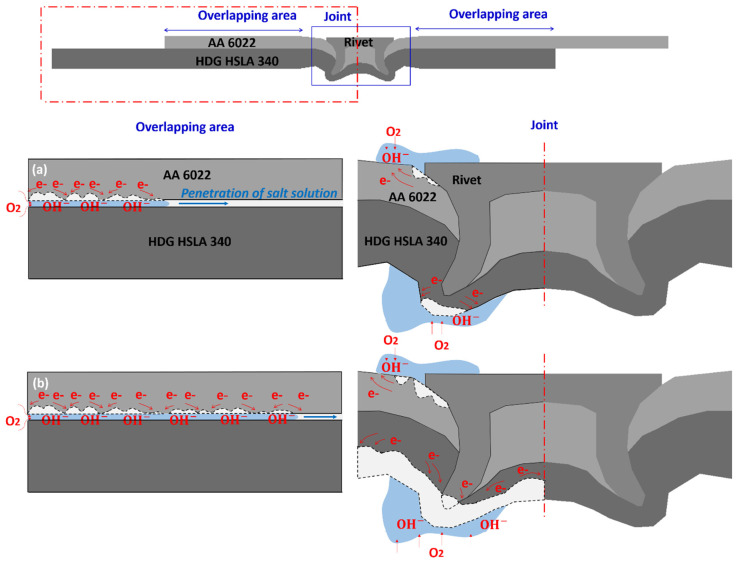
The schematic of the corrosion mechanism of SPR Al–steel joints: (**a**) Slight corrosion (26~72 cycles) and (**b**) severe corrosion (≥104 cycles).

**Figure 20:**
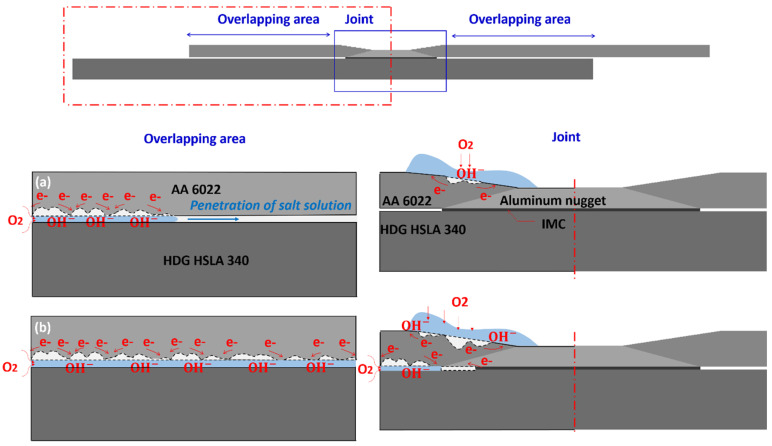
The schematic of the corrosion mechanism of RSW Al–steel joints: (**a**) Slight corrosion (14~48 cycles) and (**b**) severe corrosion (72~104 cycles).

According to the above analysis and discussion, the corrosion mechanism of SPR and RSW Al–steel joints can be concluded, as shown in Figure 19 and Figure 20, respectively.

For SPR joints, galvanic corrosion occurs at the Al–rivet head coupling area and Al–steel overlapping area by attacking AA 6022. The corrosion leaves a large number of pits on the AA 6022 surface, which affects the stiffness of the specimen significantly. The strength does not decrease since the interlock has not been affected. The interlock will be damaged under more severe corrosion (≥104 cycles) in which the rivet will be exposed and corroded.

For RSW joints, corrosion initiates on the rings of AA 6022 (indentation area). However, as corrosion proceeds, corrosion products covered on the surface prevent the salt solution from penetrating into the remaining AA 6022 (passive film). The detrimental corrosion is the galvanic corrosion that occurs at the Al–steel faying interface. The solution penetrates into the overlapping area by attacking AA 6022 first, and when the corrosion solution penetrates into the joint, SSC occurs. As a result, the strength decreases significantly. Similar to SPR joints, pits are formed in AA 6022 at the overlapping area, resulting in a decrease in the joint stiffness. Since the crevice at the Al–steel interface is much larger, galvanic corrosion here in RSW joints is much stronger than that in SPR joints, and hence, the stiffness reduction is higher.

Overall, SPR performs better at delaying the corrosion attack within the joints, hence retaining the strength, stiffness, and energy absorbed at failure.

## 4. Conclusions

In this paper, the corrosion resistance of each component in SPR and RSW joints was analyzed based on the electrochemical measurements, and the corrosion evolution and the impact on the mechanical performance were investigated.

In SPR joints, localized corrosion was first observed in the Al–rivet head interface, and up to 104 cycles, slight localized corrosion in the Al–steel interface was observed. The small crevice of the Al–steel interface retards the corrosion and maintains the joint strength. However, in RSW joints, corrosive solution penetrates into the Al–steel interface much faster, and SSC occurs, resulting in decreasing strength. The corrosion products collected at the overlap ends increased the stiffness at the beginning; however, as a large amount of material was corroded away, especially AA 6022, which became highly porous under strong galvanic corrosion occurring in the overlapping area, the stiffness decreased.

Based on the observations and analysis, the following conclusions can be drawn as listed below according to this section:

(1) Galvanic corrosion attacks AA 6022 at the overlapping area, and the most damaged zone attacked is AA 6022 at the overlap end, leaving large amounts of pits there, which decreases the joints’ stiffness significantly. The corrosion in AA 6022 propagates through the grain boundaries (IGC);
(2) In SPR joints, the detrimental corrosion occurs when the steel is corroded and then the rivet is exposed, which will result in the decrease of the mechanical interlock and the mechanical performance; while in RSW joints, the detrimental corrosion attack is along the Al–steel interface and joints fail due to SSC;
(3) Stress relaxation softens the joints and decreases the stiffness. Corrosion products collecting at the crevice help tighten the joints, which can increase the strength and the stiffness slightly;
(4) The corrosion resistance of SPR joints is much better than that of RSW joints, including retaining the strength, stiffness, and energy absorbed at failure. The slower decrease of stiffness of the F-D curve indicates lower porosity of AA 6022 at the overlapping area and a lower level of galvanic corrosion attack.

## Figures and Tables

**Table 1:** Riveting parameters of the SPR process.

Riveting Settings	Parameters
Rivet geometry	Nominal diameter: 5 mm;
	Under-head shank length: 5 mm;
	Type: countersunk;
	Material: Carbon steel and coated with zinc
Velocity	315 mm/s

**Table 2:** Welding parameters of the RSW process.

Welding Settings	Parameters
Welding force	4 KN
Squeezing time	250 ms
Pre-heating Stage	40 ms @7 KA, 10 ms cool
Welding Stage 1	250 ms @12 KA, 250 ms cool
Welding Stage 2	975 ms @13.4 KA
Holding time	250 ms

**Table 3:** Cyclic salt spray exposure (GMW14872).

Stages	Temperature	Humidity	Duration
Ambient Stage	25 ± 3 °C	~45 ± 10% RH	8 h
Humid Stage	49 ± 2 °C	~100% RH	8 h
Dry-off Stage	60 ± 2 °C	≤30% RH	8 h

**Table 4:** Chemical composition of salt spray.

Composition	Content (wt%)
NaCl	0.9
CaCl_2_	0.1
NaHCO_3_	0.075
H_2_O	98.925

**Table 5:** Settings of the rolling process.

Rolling Settings	Parameters
Diameter of the roller	127 mm
Rolling speed	20 mm/s
Thickness reduction	AA 6022: 41.7%; HSLA 340: 20%, 60%

## Data Availability

The original contributions presented in the study are included in the article, further inquiries can be directed to the corresponding author.

## Nomenclature

Δx	The displacement of crosshead;
$L_{1}$	The length of steel from the fixed end to the center of the joint;
$L_{2}$	The length of Al from moving end to the center of the joint;
$L_{12}$	The length of steel from moving end to the center of the joint when the displacement of the crosshead is Δx;
$L_{22}$	The length of Al from moving end to the center of the joint when the displacement of the crosshead is Δx;
${L^{\prime }}_{1}$	The length of steel from the fixed end to the left overlap end;
${L^{\prime }}_{2}$	The length of Al from moving end to the right overlap end;
${L^{\prime }}_{12}$	The length of steel from the fixed end to the left overlap end when the displacement of the crosshead is Δx;
${L^{\prime }}_{22}$	The length of Al from the fixed end to the left overlap end when the displacement of the crosshead is Δx;
$t_{1}$	The thickness of steel;
$t_{2}$	The thickness of Al;
d	The overlap distance;
$F_{0}$	The clamping force before loading;
F	The force applied to pull the as-received joint at a specific displacement of Δx;
$F^{\prime }$	The force applied to pull the corroded joint at a specific displacement of Δx;
$M_{1}$	The bending moment of steel;
$M_{2}$	The bending moment of Al;
θ	The rotation angle of material;
$A_{\mathrm{steel}}$	The cross-section area of steel;
$A_{\mathrm{Al}}$	The cross-section area of Al.
